# Effects of dextromethorphan on MDMA-induced serotonergic aberration in the brains of non-human primates using [^123^I]-ADAM/SPECT

**DOI:** 10.1038/srep38695

**Published:** 2016-12-12

**Authors:** Kuo-Hsing Ma, Tsung-Ta Liu, Shao-Ju Weng, Chien-Fu F. Chen, Yuahn-Sieh Huang, Sheau-Huei Chueh, Mei-Hsiu Liao, Kang-Wei Chang, Chi-Chang Sung, Te-Hung Hsu, Wen-Sheng Huang, Cheng-Yi Cheng

**Affiliations:** 1 Department of Biology and Anatomy, National Defense Medical Center, Taipei, Taiwan; 2 Graduate Institute of Life Sciences, National Defense Medical Center, Taipei, Taiwan; 3 Department of Biochemistry, National Defense Medical Center, Taipei, Taiwan; 4 Institute of Nuclear Energy Research, Taoyuan, Taiwan; 5 Department of Nuclear Medicine, Taipei Veterans General Hospital, Taipei, Taiwan; 6 Department of Nuclear Medicine, Tri-Service General Hospital, National Defense Medical Center, Taipei, Taiwan

## Abstract

3,4-Methylenedioxymethamphetamine (MDMA), a common recreational drug, is known to cause serotonergic neurotoxicity in the brain. Dextromethorphan (DM) is a widely used antitussive reported to exert anti-inflammatory effect *in vivo*. In this study, we examined the long-term effect of MDMA on the primate serotonergic system and the protective property of DM against MDMA-induced serotonergic abnormality using single photon emission computed tomography (SPECT). Nine monkeys (*Macaca cyclopis*) were divided into three groups, namely control, MDMA and co-treatment (MDMA/DM). [^123^I]-ADAM was used as the radioligand for serotonin transporters (SERT) in SPECT scans. SERT levels of the brain were evaluated and presented as the uptake ratios (URs) of [^123^I]-ADAM in several regions of interest of the brain including midbrain, thalamus and striatum. We found that the URs of [^123^I]-ADAM were significantly lower in the brains of MDMA than control group, indicating lower brain SERT levels in the MDMA-treated monkeys. This MDMA-induced decrease in brain SERT levels could persist for over four years. However, the loss of brain SERT levels was not observed in co-treatment group. These results suggest that DM may exert a protective effect against MDMA-induced serotonergic toxicity in the brains of the non-human primate.

3,4-methylenedioxymethamphetamine (MDMA, ecstasy) is a ring-substituted amphetamine derivative. It has attracted a great deal of media attention in the recent years due to its widespread abuse as a recreational drug by young people^1^. It has been reported that approximately 13 million adults aged 18 to 25 reported ecstasy use at least once in their lifetime in 2012 and there are 869,000 new users of ecstasy aged 12 and older in the USA^2^. Recent studies have shown that MDMA chronically inhibits neocortical serotonin signaling in parallel with hyperthermia^3^,^4^,^5^ and causes deficits in prospective memory and/or social intelligence^6^. Animal studies have demonstrated that repeated administration of MDMA results in clusters of aberrant serotonergic fibers^7^ and affects the serotonergic neurotransmitter system in an association with reduced density of cerebral serotonin transporters (SERT) in the brain^8^,^9^. Regarding serotonergic pathway of brain, MDMA alters serotonin (5-HT), 5-HT receptors, and SERT levels^9^,^10^. MDMA is known to have a high affinity to α-adrenergic and nicotinic receptors (e.g. NMDA receptor)^11^. It has been reported that blocking of SERT or activation of NMDA receptor by MDMA cause the death of neurons^12^,^13^.

The involvement of SERT in the pathological mechanism of MDMA has been well documented, along with therapeutic drugs to protect against MDMA-induced neurotoxicity^9^,^14^,^15^,^16^. Dextromethorphan (DM, 3-methoxy-17-methylmorphinan) is a typical morphine like opioid, which is derived from levorphanol^17^. DM is known for its acceptable safety and efficacy profiles with no sedative or addictive properties at recommended antitussive doses^18^,^19^. It is also a relatively selective and specific NMDA receptor antagonist and reported to inhibit serotonin uptake into neurons^20^,^21^,^22^,^23^. Base on its pharmacological effects, DM is used for treating pseudobulbar affect (PBA, a common manifestation of brain pathology associated with many neurological diseases)^24^ and prescribed as a neuroprotective agent for seizures, cerebral ischemia and Parkinson’s disease^19^. Although DM have been reported for its protect effect on methamphetamine-induced neurotoxicity *in vivo*^25^, it remains unclear whether DM has neuroprotective effects against MDMA-induced damage in cerebral SERT changes.

^123^I-ADAM {2-((2-((dimethylamino)methyl)phenyl)thio)-5- [^123^I]iodophenylamine} is a SPET tracer that has shown a high binding affinity for SERT and has been proven to have excellent brain uptake in rats^26^. Newberg *et al*. have suggested that used [^123^I]-ADAM to demonstrate decreased SERT binding in midbrain of patients with major depression^27^. In our previous studies, [^123^I]-ADAM was used to monitor the serotonergic system in brain of non-human primate^28^,^29^. We also found that MDMA induced neurite damage and neuron death in serotonergic neuron *in vitro*^30^. Moreover, resveratrol, a natural polyphenolic phytoalexin, could protected against MDMA-induced decrease in SERT availability in midbrain and thalamus of rats^9^. In the present study, SERT density of different brain regions of the monkey *(macaca cyclopis*) were assessed based on the uptakes of [^123^I]-ADAM using single photon emission computerized tomography (SPECT). Dynamic imaging of SPECT was performed to evaluate protective effects of DM against MDMA-induced damage in the serotonergic system, which is associated with density of SERT of the monkey brains.

## Results

### MRI, SPECT images and [^123^I] -ADAM Uptake ratios (URs)

**Figure 1:**
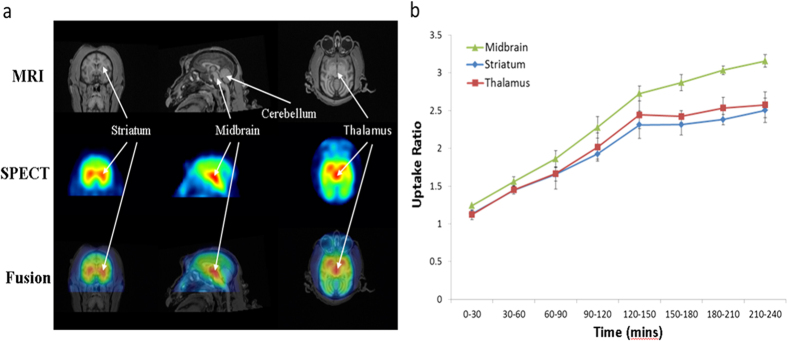
(**a**) Representative images of MRI and [^123^I]-ADAM/SPECT in coronal (left column), sagittal (middle column) and horizontal (right column) views. (**b**) Uptake ratios of [^123^I]-ADAM in the midbrain, striatum and thalamus at different time points in normal monkeys. The data are expressed as the mean ± standard deviation (S.D.).

The representative MR images of and [^123^I]-ADAM distributions in the midbrain, striatum, thalamus and cerebellum of normal healthy monkey brain were shown in Fig. 1a. URs of [^123^I]-ADAM over time in striatum, thalamus and midbrain of normal monkeys were analyzed and shown in Fig. 1b. The highest uptake was found in the midbrain followed by the thalamus and striatum. There was almost no radioactive uptake in the cerebellum.

### MDMA administration causes serotonergic abnormality

**Figure 2:**
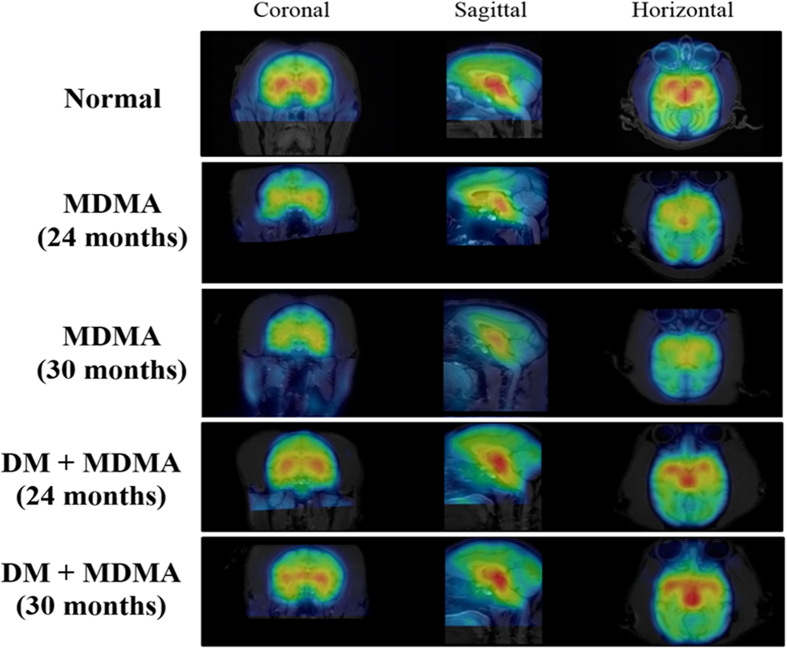
Representative MRI and [^123^I]-ADAM SPECT fusion images in coronal, sagittal and horizontal views at different time points (24 and 30 months) in normal, MDMA and DM+MDMA groups.

**Figure 3:**
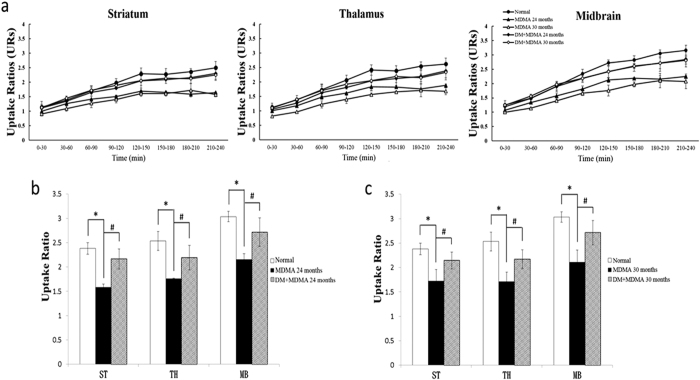
(**a**) Time-course of mean URs in striatum, thalamus and midbrain at different time points in normal, MDMA and DM+MDMA groups. (**b**) URs of [^123^I]-ADAM SPECT during 210–240 min post-injection in various brain regions of normal, MDMA DM+MDMA groups at 24 months. These results are presented as mean ± S.D. *P < 0.05. compared with normal group; ^#^P < 0.05. compared with MDMA group. (**c**) URs of [^123^I]-ADAM SPECT during 210–240 min. in various brain regions of normal, MDMA and DM+MDMA groups at 30 months. These results are presented as mean ± S.D. *P < 0.05. compared with normal group; ^#^P < 0.05. compared with MDMA group.

To assess the influence of MDMA on serotonergic system, [^123^I]-ADAM SPECT scan was carried out in the monkeys after MDMA administration. As shown in Fig. 2, the SPECT images of the brain of MDMA-treated monkey showed significantly decreased uptakes of [^123^I]-ADAM in the striatum, thalamus and midbrain compared with those of normal monkeys. The mean URs of [^123^I]-ADAM in the three regions after MDMA treatment increased over time post-injection and gradually reached a plateau at 210–240 min (Supplementary Fig. S1a–c). MDMA treated monkeys exhibited significantly lower URs of [^123^I]-ADAM at 24 months (1.64 ± 0.03 in striatum, 1.88 ± 0.05 in thalamus and 2.24 ± 0.03 in midbrain, respectively) compared with those of normal monkeys (2.50 ± 0.22 in striatum, 2.62 ± 0.21 in thalamus and 3.16 ± 0.17 midbrain, respectively) (Fig. 3). This MDMA- induced serotonergic abnormality had last for up to 54 months (Supplementary Fig. S1d).

### Treatment of DM restored MDMA-induced abnormality in monkey brain

We next determined the effect of DM on serotonergic abnormality induced by MDMA. [^123^I]-ADAM uptake distributions of monkeys co-treated with MDMA and DM were similar to those of the normal group (Fig. 2). Quantitative analysis revealed that mean URs of the regions of interest in DM+MDMA monkeys at 24 months were 2.30 ± 0.19 in striatum, 2.38 ± 0.23 in thalamus and 2.85 ± 0.26 in midbrain, respectively, similar to the normal URs (Fig. 3). Therefore, the MDMA-induced serotonergic abnormality can be abolished by DM and this effect can last for up to 30 months (the mean URs were 2.25 ± 0.18 in striatum, 2.34 ± 0.27 in thalamus and 2.81 ± 0.13 in midbrain, respectively) (Supplementary Fig. S2).

## Discussion

In the present study, we demonstrated that MDMA induced long-term serotonergic lesions in the brain of macaques on [^123^I]-ADAM/SPECT scan. We showed that co-treatment with DM abolished MDMA-induced aberration in SERT density in several brain regions of the non-human primate.

In rodents, exposure to MDMA repeatedly have been shown to produce deficits in the brain causing neuronal death or memory loss^31^,^32^,^33^,^34^,^35^. Callahan and his colleagues found MDMA causes structural damage to axonal transport mechanisms in multiple brain regions^36^. Moreover, Schouw *et al*. observed a reduction in hemodynamic response in a brain region with reduced SERT densities by using pharmacological MRI, presumably reflecting serotonin mediated changes in neuronal activity^37^. In our study, the URs of [^123^I]-ADAM in various brain regions of MDMA-treated group were significantly lower than those of normal controls suggested the damage might happened and caused density of cerebral SERT decreasing in the serotonergic neurotransmitter system. In addition, [^123^I]-ADAM SPECT co-registration with MRI is well suited for *in vivo* assessment of MDMA-induced damage in the brain of non-human primate. Also, effects of MDMA on brain in nonhuman primate were reported harmfully. Ricaurte and his colleague suggested that squirrel monkeys showed serotonergic deficits after MDMA treatment (5 mg/kg, s.c. twice daily for 4 consecutive days) and reduction of 5-HT, 5-HIAA, and [^3^H] paroxetine were observed 18 months after administration of MDMA^38^. When used the same dosage of MDMA in baboon, Scheffel and his colleague observed that PET imaging with [^11^C](+)McN 5652 as the radioligand could detect the reduction in SERT density secondary to MDMA-induced neurotoxicity^39^. Furthermore, Reneman and his colleague found that treated with MDMA which following previous studies caused SERT density reduced by 39% in hypothalamic/midbrain region of rhesus monkey brain when using [^123^I]β-CIT SPECT scan^40^. However, those studies didn’t investigate the drug effect for a long time after MDMA treatment. Based on our results, we also found that MDMA-induced damage which is associated with reduced density of cerebral SERT and this serotonergic aberration could persist over four years in the brain of non-human primate.

Dextromethorphan (DM) is a non-opioid morphinan derivative that has been used safely and extensively as an antitussive drug for about 50 years. Also, many preclinical evidences of neuroprotetive properties of DM *in vitro* and *in vivo* were reported^41^,^42^,^43^,^44^. *In vivo* models of ischemic brain injury have shown that DM protects the brain against infarction and functional consequences of injury^45^,^46^. *In vitro*, Liu *et al*. reported that DM significantly attenuates the lipopolysaccharide-induced reduction the number of dopaminergic neuron and protects against inflammation-mediated degeneration^47^. Accordingly, in our studies, DM can prevent MDMA-induced serotonergic abnormity which is associated with reduced density of cerebral SERT and the protective effect will observed till to 2.5 years. Furthermore, including the striatum, thalamus and midbrain, all have the restoration phenomenon in DM treatment suggest that the protective mechanism may be through maybe increasing blood flow or reducing of cell death^48^,^49^. DM protects against brain injury by induced the c*-fos* protein in the core territory of the middle cerebral artery and reduced the neuronal edema and necrosis in the hippocampus^50^. Posod *et al*. provide evidence indicating that DM treatment prevents caspase-3 activation and increased the cell viability in hyperoxia-induced cell death^48^.

Moreover, DM inhibits the NMDA receptor^51^. Collins and his colleagues found MDMA-induced loss of parvalbumin interneurons is mediated by NMDA and 5HT2A receptors^52^. It has been shown that NMDA receptor activation inhibits the actin-based protrusive activity of dendritic spines^53^. Those results provided a possible protective mechanism of DM (antagonist of NMDA receptor may promote or allow dendritic spine maintenance) in our MDMA-induced monkey model. Also, DM has been reported to increase serotonin levels^54^, through activation of sigma-1 receptors, which has been shown to modulate monoamine neurotransmitter levels^55^,^56^,^57^. This data suggested block of MDMA in DA and SERT will be compensated possibly by DM triggered release. In this study, the URs of the co-treatment (MDMA/DM) group showed no significant difference when compared to the normal control data, but obviously higher than those of MDMA-treated group. These results indicate that MDMA-induced serotonergic abnormality persisted for over four years in the non-human primate and the DM could alleviate this abnormity.

A number of studies have suggested that SERT is highly involved in MDMA-induced neurotoxicity^13^,^58^,^59^,^60^. The binding of MDMA to SERT of the 5-HT nerve terminal leads to the release of 5-HT from the storage vesicles^61^ and causes an acute increase in 5-HT level in the pre-synaptic neuron. Thus, MDMA can trigger a rapid pre-synaptic accumulation of hydrogen peroxide, a product of 5-HT metabolism involving monoamine oxidase B (MAO-B)^13^,^62^. Hydrogen peroxide can be converted into hydroxyl radical to cause oxidative stress in mitochondria of the serotonergic neurons^9^,^13^,^63^. Therefore, a potential therapeutic strategy against MDMA-induced serotonergic neurotoxicity is using selective serotine reuptake inhibitors (SSRIs) to prevent MDMA from entering the pre-synaptic terminals^64^. DM could act like SSRIs to inhibit the binding of MDMA to SERT, thereby exhibiting a protective effect against MDMA-induced neurotoxicity^65^.

Taken together, we showed that MRI coupled with [^123^I]-ADAM SPECT image is well suited for *in vivo* assessment of serotonergic neurotransmitter system. The neurotoxicity accompanied with reduced density of SERT by acute MDMA administration will observed for 4.5 years in the monkey brain and treatment of DM can rescue this phenomenon in different brain region. Further studies evaluating DM’s usefulness in clinical setting are direly needed.

## Methods

### Animals

Nine Formosan rock monkeys (*Macaca cyclopis*, weight about 5–8.5 kg) were housed under constant temperature, humidity, and a 12-h light/dark cycle in the Laboratory Animal Center of National Defense Medical Center (NDMC), which is accredited by the Association for Assessment and Accreditation of Laboratory Animal Care, International (AAALAC International). The basic information of the monkeys such as body weight and temperature were shown in Supplementary Tables S1 and S2. These measurements were provided by animal center of NDMC annually. Study protocols were approved National Defense Medical Center Animal Care and Use Committee and were conducted in compliance with the guidelines of National Institute of Health for care and use the laboratory animals (IACUC approval No.: IACUC-11-234).

### Experimental procedures

**Figure 4:**
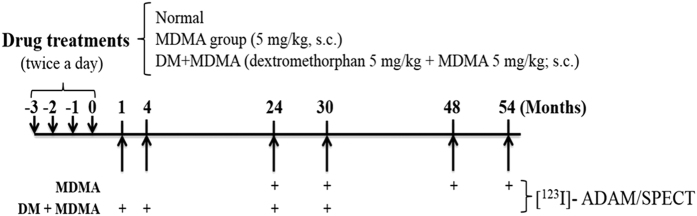
Experimental flow chart of study. “+” indicates a performance of [^123^I]-ADAM/SPECT. “s.c.” indicates subcutaneous injection.

The experimental design was shown in Fig. 4. Briefly, MDMA (purity: 98%, Investigation Bureau of Taiwan) and DM (Sigma-Aldrich) were dissolved in saline (0.9% NaCl) for subcutaneous (s.c.) injection. Monkeys were fasted overnight and anesthetized with ketamine (10 mg/kg), followed by passive inhalation of oxygen with 1.8% isoflurane at a flow rate of 2 L/min (keeping oxygen saturation 99.5%). For radiotracer administration, intravenous infusion via the cephalic vein was used with 0.9% NaCl at a flow rate of 5 mL/kg/h. Potassium perchlorate (200 mg) was administered orally 20 min prior to radiotracer injection in order to minimize ^123^I uptake by the thyroid. In the experiments, [^123^I]-ADAM protocols were performed at least approximately 4-week intervals. All drugs were administered twice daily for 4 consecutive days as previously described^9^. For DM+MDMA group, DM (5 mg/kg; s.c.) and MDMA (5 mg/kg; s.c.) were given to the animals twice daily (at 9:00 AM and 5:00 PM) for four consecutive days (Fig. 4) while DM was given five minutes ahead of MDMA administration. In order to evaluate the long-term effects of MDMA/DM, SERT imaging were measured at 1, 4, 24, 30, 48 and 54 months respectively.

### Synthesis of SPECT ligands

Radiosynthesis of [^123^I]ADAM was performed at Institute of Nuclear Energy Research, Lung-Tan, Taiwan. The ^123^I-labelled ADAM was prepared by iododestannylation of tin precursor (100 mg), with carrier-free ^123^I as NaI (approximately 5.55GBq) in the presence of hydrogen peroxide in dilute acetic acid. 5 min later, the reaction was quenched with NaHSO_3_. In order to perform the neutralization, the reaction solution was loaded on an octyl cartridge (Accubond, J&W Scientific, Folsom, CA, USA) and eluted in turn. The injection solution was dissolved in 50% (v/v) ethanol. Purified [^123^I] -ADAM was collected by eluting from the cartridge with absolute ethanol, and then diluted with 0.9% saline solution to a specific activity of greater than 4.44 × 10^5^ GBq/mmole (12,000 Ci/mmole).

The radiochemical purity of the solution was always higher than 90%, as determined by high-pressure liquid chromatography (HPLC) on a Hamilton PRP-1 column (4.1 × 250 mm; Hamilton Co. Reno, NV, USA). Elution was by means of isocratic acetonitrile/5 mM dimethyl glutaric acid (DMGA) (pH 7.0) 90:10 solution at a flow rate of 1 ml/min.

### SPECT analysis in monkey brain

185 MBq (5 mCi) of ^123^I-ADAM was administered and then SPECT was started immediately. Brain imaging of SPECT was performed on a rotating camera equipped with ultra-high resolution fan-beam collimators (Hawkeye, Millennium VG, General Electric Medical Systems, Milwaukee, WI, USA). Spatial resolution of this equipment has a 11.2 mm in full-width at half-maximum at 10 cm from the collimator face. A 10% symmetric window was used for [^123^I]-ADAM (143–175 keV).

The image data were corrected for photon attenuation based on the protocol of the previous study with minor modification^29^. Briefly, SPECT image was created by reconstructing the projection data from every scan per 30 min over the full 4-h course of the experiment using filtered back-projection with a Metz filter. The data were corrected for photon attenuation using Chang’s first order method. All the SPECT data were acquired by the same investigator. Regions of interest (ROIs) were drawn in reference to a corresponding magnetic resonance imaging (MRI) of the midbrain (MB), thalamus (TH), striatum (ST) and cerebellum (CB). The MRI images were resliced, resized, and coregistered to all corresponding SPECT images in planes parallel to the canthomeatal line. The image data analysis and radioactivity of [^123^I]-ADAM in monkeys’ brain were analyzed by Pmod 3.1 software (PMOD Technologies, Switzerland). The uptake ratios (URs) were calculated as follows: UR = mean counts per pixel in the target area (MB, TH or ST)/mean counts per pixel in the CB.

### MRI scanning

T2 Weighted MR Imaging (T2WI) was performed at a 3.0 Tesla GE SIGNA 450 system. After three-plane tripilot imaging, 22 contiguous coronal, sagittal and horizontal T2WIs were acquired using a fast spin echo sequence with TR/TE = 3000/100 ms, echo train length = 8, NEX = 4, matrix size = 256 × 256, FOV = 20 × 20 mm^2^, slice thickness (SLTH) = 4 mm, flip angle = 90°, bandwidth = 50.0 kHz, and acquisition time = 6 min 44 s. All animals in our study were acquired with the same MR parameters. After the image acquisition was completed, images were transferred to a stand-alone personal computer and using N.I.H. Image 1.52 software for analysis. The MR image were then resliced, resized and co-registered to all corresponding SPECT images in plane parallel to the canthomeatal line (CML).

### Statistical analysis

One-way ANOVA was performed for regions of interest (ROI) data (LABCAT In-Life v. 6.2). When appropriate, a post-hoc analysis (Dunnett’s or tukey’s t-test) was carried out. In all cases, statistical significance was defined as p ≤ 0.05.

## Additional Information

**How to cite this article**: Ma, K.-H. *et al*. Effects of dextromethorphan on MDMA-induced serotonergic aberration in the brains of non-human primates using [^123^I]-ADAM/SPECT. *Sci. Rep.* **6**, 38695; doi: 10.1038/srep38695 (2016).

**Publisher's note:** Springer Nature remains neutral with regard to jurisdictional claims in published maps and institutional affiliations.

## Supplementary Material

Supplementary Dataset 1

## References

[b1] Schmid Y. . Effects of methylphenidate and MDMA on appraisal of erotic stimuli and intimate relationships. European Neuropsychopharmacology 25, 17–25 (2015). 25498417 10.1016/j.euroneuro.2014.11.020

[b2] Carrera P. & Iyer V. N. Profound hypoglycemia with ecstasy intoxication. Case Reports in Emergency Medicine 2015, 483153, doi: 10.1155/2015/483153 (2015). 25692049 PMC4322300

[b3] Benningfield M. M. & Cowan R. L. Brain serotonin function in MDMA (ecstasy) users: evidence for persisting neurotoxicity. Neuropsychopharmacology 38, 253–255 (2013). 23147495 10.1038/npp.2012.178 PMC3521982

[b4] Parrott A. C. Human research on MDMA (3,4-methylene- dioxymethamphetamine) neurotoxicity: cognitive and behavioural indices of change. Neuropsychobiology 42, 17–24 (2000). 10867552 10.1159/000026666

[b5] Parrott A. C. MDMA and temperature: a review of the thermal effects of ‘Ecstasy’ in humans. Drug and Alcohol Dependence 121, 1–9 (2012). 21924843 10.1016/j.drugalcdep.2011.08.012

[b6] Parrott A. C. Human psychobiology of MDMA or ‘Ecstasy’: an overview of 25 years of empirical research. Human Psychopharmacology 28, 289–307 (2013). 23881877 10.1002/hup.2318

[b7] Adori C. . Recovery and aging of serotonergic fibers after single and intermittent MDMA treatment in Dark Agouti rat. Journal of Comparative Neurology 519, 2353–2378 (2011). 21456018 10.1002/cne.22631

[b8] Parrott A. C. MDMA, serotonergic neurotoxicity, and the diverse functional deficits of recreational ‘Ecstasy’ users. Neuroscience & Biobehavioral Reviews 37, 1466–1484 (2013). 23660456 10.1016/j.neubiorev.2013.04.016

[b9] Shih J. H. . Evaluation of brain SERT occupancy by resveratrol against MDMA-induced neurobiological and behavioral changes in rats: A 4-[(18)F]-ADAM/small-animal PET study. European Neuropsychopharmacology 26, 92–104 (2016). 26612383 10.1016/j.euroneuro.2015.11.001

[b10] Lizarraga L. E. . Vesicular monoamine transporter 2 and the acute and long-term response to 3,4-(±)-methylenedioxymethamphetamine. Toxicological Sciences 143, 209–219 (2015). 25370842 10.1093/toxsci/kfu222 PMC4274386

[b11] Schenk S. MDMA (“ecstasy”) abuse as an example of dopamine neuroplasticity. Neuroscience & Biobehavioral Reviews 35, 1203–1218 (2011). 21184779 10.1016/j.neubiorev.2010.12.010

[b12] Capela J. P. . Ecstasy-induced cell death in cortical neuronal cultures is serotonin 2A-receptor-dependent and potentiated under hyperthermia. Neuroscience 139, 1069–1081 (2006). 16504407 10.1016/j.neuroscience.2006.01.007

[b13] Hrometz S. L. . 3,4-methylenedioxymethamphetamine (MDMA, ecstasy)-mediated production of hydrogen peroxide in an *in vitro* model: the role of dopamine, the serotonin-reuptake transporter, and monoamine oxidase-B. Neuroscience Letters 367, 56–9 (2004). 15308297 10.1016/j.neulet.2004.05.075

[b14] Lanteri C. . Repeated exposure to MDMA triggers long-term plasticity of noradrenergic and serotonergic neurons. Molecular Psychiatry 19, 823–33 (2014). 23958955 10.1038/mp.2013.97

[b15] Malberg J. E., Sabol K. E. & Seiden L. S. Co-administration of MDMA with drugs that protect against MDMA neurotoxicity produces different effects on body temperature in the rat. Journal of Pharmacology and Experimental Therapeutics 278, 258–267 (1996). 8764359

[b16] Morley K. C. . Cannabinoids prevent the acute hyperthermia and partially protect against the 5-HT depleting effects of MDMA (“Ecstasy”) in rats. Neuropharmacology 46, 954–965 (2004). 15081792 10.1016/j.neuropharm.2004.01.002

[b17] Nguyen L. . Dextromethorphan: An update on its utility for neurological and neuropsychiatric disorders. Pharmacology & Therapeutics 159, 1–22 (2016). 26826604 10.1016/j.pharmthera.2016.01.016

[b18] Bem J. L. & Peck R. Dextromethorphan. An overview of safety issues. Drug Safety. 7, 190–199 (1992). 1503667 10.2165/00002018-199207030-00004

[b19] Shin E. J. . Neuropsychotoxic and neuroprotective potentials of dextromethorphan and its analogs. Journal of Pharmacological Sciences 116, 137–148 (2011). 21606622 10.1254/jphs.11r02cr

[b20] Musacchio J. M., Klein M. & Canoll P. D. Dextromethorphan sites, sigma receptors, and the psychotomimetic effects of sigma opiates. Progress in Clinical & Biological Research 328, 13–16 (1990). 2154766

[b21] Steinberg G. K. . Dextromethorphan protects against cerebral injury following transient focal ischemia in rabbits. Stroke 19, 1112–1118 (1988). 3413809 10.1161/01.str.19.9.1112

[b22] Tortella F. C. . Autoradiographic localization of 3H-dextromethorphan binding sites differs from NMDA. NIDA-Research Monographs 95, 548–549 (1989). 2561854

[b23] Tortella F. C., Pellicano M. & Bowery N. G. Dextromethorphan and neuromodulation: old drug coughs up new activities. Trends in Pharmacological Sciences 10, 501–507 (1989). 2694543 10.1016/0165-6147(89)90050-3

[b24] Cummings J. L. . Defining and diagnosing involuntary emotional expression disorder. CNS Spectrums 11, 1–7 (2006). 10.1017/s1092852900026614 16816786

[b25] Thomas D. M. & Kuhn D. M. MK-801 and dextromethorphan block microglial activation and protect against methamphetamine-induced neurotoxicity. Brain Research 1050, 190–198 (2005). 15987631 10.1016/j.brainres.2005.05.049

[b26] Oya S. . 2-((2-((dimethylamino)methyl)phenyl)thio)-5-iodophenylamine (ADAM): an improved serotonin transporter ligand. Nuclear Medicine and Biology 27, 249–254 (2000). 10832081 10.1016/s0969-8051(00)00084-6

[b27] Newberg A. B. . ^123^I-ADAM binding to serotonin transporters in patients with major depression and healthy controls: a preliminary study. Journal of Nuclear Medicine 46, 973–977 (2005). 15937308

[b28] Ma K. H. . Imaging serotonin transporters using [^123^I]ADAM SPECT in a parkinsonian primate model. Applied Radiation and Isotopes 66, 1799–1803 (2008). 18703341 10.1016/j.apradiso.2008.06.033

[b29] Ma K. H. . Simultaneous [^99m^Tc]TRODAT-1 and [^123^I]ADAM brain SPECT in nonhuman primates. Molecular Imaging and Biology 11, 253–262 (2009). 19225845 10.1007/s11307-009-0197-0

[b30] Li I. H. . Involvement of autophagy upregulation in 3,4-methylenedioxymethamphetamine (‘ecstasy’)-induced serotonergic neurotoxicity. Neurotoxicology 52, 114–126 (2016). 26610922 10.1016/j.neuro.2015.11.009

[b31] Able J. A. . 3,4-methylenedioxymethamphetamine in adult rats produces deficits in path integration and spatial reference memory. Biological Psychiatry 59, 1219–1226 (2006). 16324685 10.1016/j.biopsych.2005.09.006 PMC2888296

[b32] Cunningham J. I. . MDMA pretreatment leads to mild chronic unpredictable stress-induced impairments in spatial learning. Behavioral Neuroscience 123, 1076–1084 (2009). 19824774 10.1037/a0016716 PMC2786777

[b33] Farré M. . Human pharmacology of 3,4-methylenedioxymethamphetamine (MDMA, ecstasy) after repeated doses taken 4 h apart Human pharmacology of MDMA after repeated doses taken 4 h apart. European Neuropsychopharmacology 25, 1637–1649 (2015). 26073279 10.1016/j.euroneuro.2015.05.007

[b34] Kay C., Harper D. N. & Hunt M. The effects of binge MDMA on acquisition and reversal learning in a radial-arm maze task. Neurobiology of Learning and Memory 95, 473–483 (2011). 21371565 10.1016/j.nlm.2011.02.010

[b35] Tamburini I. . MDMA induces caspase-3 activation in the limbic system but not in striatum. Annals of the New York Academy of Sciences 1074, 377–381 (2006). 17105935 10.1196/annals.1369.037

[b36] Callahan B. T., Cord B. J. & Ricaurte G. A. Long-term impairment of anterograde axonal transport along fiber projections originating in the rostral raphe nuclei after treatment with fenfluramine or methylenedioxymethamphetamine. Synapse 40, 113–121 (2001). 11252022 10.1002/syn.1032

[b37] Schouw M. L. . Mapping serotonergic dysfunction in MDMA (ecstasy) users using pharmacological MRI. European Neuropsychopharmacology 22, 537–545 (2012). 22209360 10.1016/j.euroneuro.2011.12.002

[b38] Ricaurte G. A. . Lasting effects of (+-)-3,4-methylenedioxymethamphetamine (MDMA) on central serotonergic neurons in nonhuman primates: neurochemical observations. Journal of Pharmacology and Experimental Therapeutics 261, 616–622 (1992). 1374470

[b39] Scheffel U. . *In vivo* detection of short- and long-term MDMA neurotoxicity–a positron emission tomography study in the living baboon brain. Synapse 29, 183–192 (1998). 9593108 10.1002/(SICI)1098-2396(199806)29:2<183::AID-SYN9>3.0.CO;2-3

[b40] Reneman L. . Validity of [^123^I]beta-CIT SPECT in detecting MDMA-induced serotonergic neurotoxicity. Synapse 46, 199–205 (2002). 12325046 10.1002/syn.10130

[b41] Choi D. W., Peters S. & Viseskul V. Dextrorphan and levorphanol selectively block N-methyl-D-aspartate receptor-mediated neurotoxicity on cortical neurons. Journal of Pharmacology and Experimental Therapeutics 242, 713–720 (1987). 3039122

[b42] DeCoster M. A. . Sigma receptor-mediated neuroprotection against glutamate toxicity in primary rat neuronal cultures. Brain Research 671, 45–53 (1995). 7728532 10.1016/0006-8993(94)01294-r

[b43] Shin E. J. . Neuropsychotoxicity of abused drugs: potential of dextromethorphan and novel neuroprotective analogs of dextromethorphan with improved safety profiles in terms of abuse and neuroprotective effects. Journal of Pharmacological Sciences 106, 22–27 (2008). 18198471 10.1254/jphs.fm0070177

[b44] Trube G. & Netzer R. Dextromethorphan: cellular effects reducing neuronal hyperactivity. Epilepsia 35, S62–S67 (1994). 7518769 10.1111/j.1528-1157.1994.tb05972.x

[b45] Kim H. C. . Anticonvulsant effects of new morphinan derivatives. Bioorganic & Medicinal Chemistry Letters 11, 1651–1654 (2001). 11425529 10.1016/s0960-894x(01)00262-1

[b46] Kim H. C. . The effects of dextromethorphan on kainic acid-induced seizures in the rat. Neurotoxicology 17, 375–385 (1996). 8856734

[b47] Liu Y. . Dextromethorphan protects dopaminergic neurons against inflammation-mediated degeneration through inhibition of microglial activation. Journal of Pharmacology and Experimental Therapeutics 305, 212–218 (2003). 12649371 10.1124/jpet.102.043166

[b48] Posod A. . The common antitussive agent dextromethorphan protects against hyperoxia-induced cell death in established *in vivo* and *in vitro* models of neonatal brain injury. Neuroscience 274, 260–272 (2014). 24912029 10.1016/j.neuroscience.2014.05.059

[b49] Steinberg G. K. . Dextromethorphan alters cerebral blood flow and protects against cerebral injury following focal ischemia. Neuroscience Letters 133, 225–228 (1991). 1816501 10.1016/0304-3940(91)90575-e

[b50] Bao W. L. . Inhibitory effects of dextromethorphan on c-fos protein expression during focal cerebral ischemia in rats. Zhongguo Yao Li Xue Bao 17, 418–420 (1996). 9863163

[b51] Woo T. M. & Hanley J. R. “How high do they look?” : identification and treatment of common ingestions in adolescents. Journal of Pediatric Health Care 27, 135–144 (2013). 23414979 10.1016/j.pedhc.2012.12.002

[b52] Collins S. A., Gudelsky G. A. & Yamamoto B. K. MDMA-induced loss of parvalbumin interneurons within the dentate gyrus is mediated by 5HT2A and NMDA receptors. European Journal of Pharmacology 761, 95–100 (2015). 25936514 10.1016/j.ejphar.2015.04.035 PMC4532576

[b53] Sala C., Cambianica I. & Rossi F. Molecular mechanisms of dendritic spine development and maintenance. Acta Neurobiologiae Experimentalis (Warsaw) 68, 289–304 (2008). 10.55782/ane-2008-1696 18511962

[b54] Codd E. E. . Serotonin and norepinephrine uptake inhibiting activity of centrally acting analgesics: Structural determinants and role in antinociception. Journal of Pharmacology and Experimental Therapeutics 274, 1263–1270 (1995). 7562497

[b55] Kobayashi T. . Sigma 1 receptor subtype is involved in the facilitation of cortical dopaminergic transmission in the rat brain. Neurochemical Research 22, 1105–1109 (1997). 9251100 10.1023/a:1027361101419

[b56] Bermack J. E. & Debonnel G. Modulation of serotonergic neurotransmission by short- and long-term treatments with sigma ligands. British Journal of Pharmacology 134, 691–699 (2001). 11588125 10.1038/sj.bjp.0704294 PMC1572988

[b57] Lucas G. . Further evidence for an antidepressant potential of the selective sigma1 agonist SA 4503: Electrophysiological, morphological and behavioural studies. International Journal of Neuropsychopharmacology 11, 485–495 (2008). 18364064 10.1017/S1461145708008547

[b58] Schmidt C. J. & Taylor V. L. Reversal of the acute effects of 3,4-methylenedioxymethamphetamine by 5-HT uptake inhibitors. European Journal of Pharmacology 181, 133–136 (1990). 1974853 10.1016/0014-2999(90)90254-4

[b59] Shankaran M., Yamamoto B. K. & Gudelsky G. A. Involvement of the serotonin transporter in the formation of hydroxyl radicals induced by 3,4-methylenedioxymethamphetamine. European Journal of Pharmacology 385, 103–110 (1999). 10607865 10.1016/s0014-2999(99)00728-1

[b60] Li I. H. . Study on the neuroprotective effect of fluoxetine against MDMA- induced neurotoxicity on the serotonin transporter in rat brain using micro-PET. NeuroImage 49, 1259–1270 (2010). 19682588 10.1016/j.neuroimage.2009.07.072

[b61] Partilla J. S. . Interaction of amphetamines and related compounds at the vesicular monoamine transporter. Journal of Pharmacology and Experimental Therapeutics 319, 237–246 (2006). 16835371 10.1124/jpet.106.103622

[b62] Alves E. . Monoamine oxidase-B mediates ecstasy-induced neurotoxic effects to adolescent rat brain mitochondria. The Journal of Neuroscience 27, 10203–10210 (2007). 17881526 10.1523/JNEUROSCI.2645-07.2007 PMC6672671

[b63] Lipinski B. Hydroxyl radical and its scavengers in health and disease. Oxidative Medicine and Cellular Longevity 2011, 809696 Review. (2011). 21904647 10.1155/2011/809696 PMC3166784

[b64] Capela J. P. . Molecular and cellular mechanisms of ecstasy-induced neurotoxicity: an overview. Molecular Neurobiology 39, 210–271 (2009). 19373443 10.1007/s12035-009-8064-1

[b65] Taylor C. P. . Pharmacology of dextromethorphan: Relevance to dextromethorphan/quinidine (Nuedexta^®^) clinical use. Pharmacology & Therapeutics 164, 170–182 Review. (2016). 27139517 10.1016/j.pharmthera.2016.04.010

