# Supplementary material for: Effects of dextromethorphan on MDMA-induced serotonergic aberration in the brains of non-human primates using [123I]-ADAM/SPECT
Source: Sci Rep. 2016 Dec 12;6:38695. doi: 10.1038/srep38695 (PMC5150522; doi:10.1038/srep38695)

**Effects of dextromethorphan on MDMA-induced serotonergic aberration in the brains of non-human primates using [^123^I]-ADAM/SPECT**

Kuo-Hsing Ma ^a^, Tsung-Ta Liu ^a^, Shao-Ju Weng ^a^, Chien-Fu F. Chen ^b^, Yuahn-Sieh Huang ^a^, Sheau‐Huei Chueh ^c^, Mei-Hsiu Liao ^d^, Kang-Wei Chang ^d^, Chi-Chang Sung ^a^, Te-Hung Hsu ^a^, Wen-Sheng Huang ^e^, Cheng-Yi Cheng ^f^, *

^a^ Department of Biology and Anatomy, National Defense Medical Center, Taipei, Taiwan

^b^ Graduate Institute of Life Sciences, National Defense Medical Center, Taipei, Taiwan

^c^ Department of Biochemistry, National Defense Medical Center, Taipei, Taiwan

^d^ Institute of Nuclear Energy Research, Taoyuan, Taiwan

^e^ Department of Nuclear Medicine, Taipei Veterans General Hospital, Taipei, Taiwan.

^f^ Department of Nuclear Medicine, Tri-Service General Hospital, National Defense Medical Center, Taipei, Taiwan

*Corresponding author : Cheng-Yi Cheng

**Supplementary Figure S1**


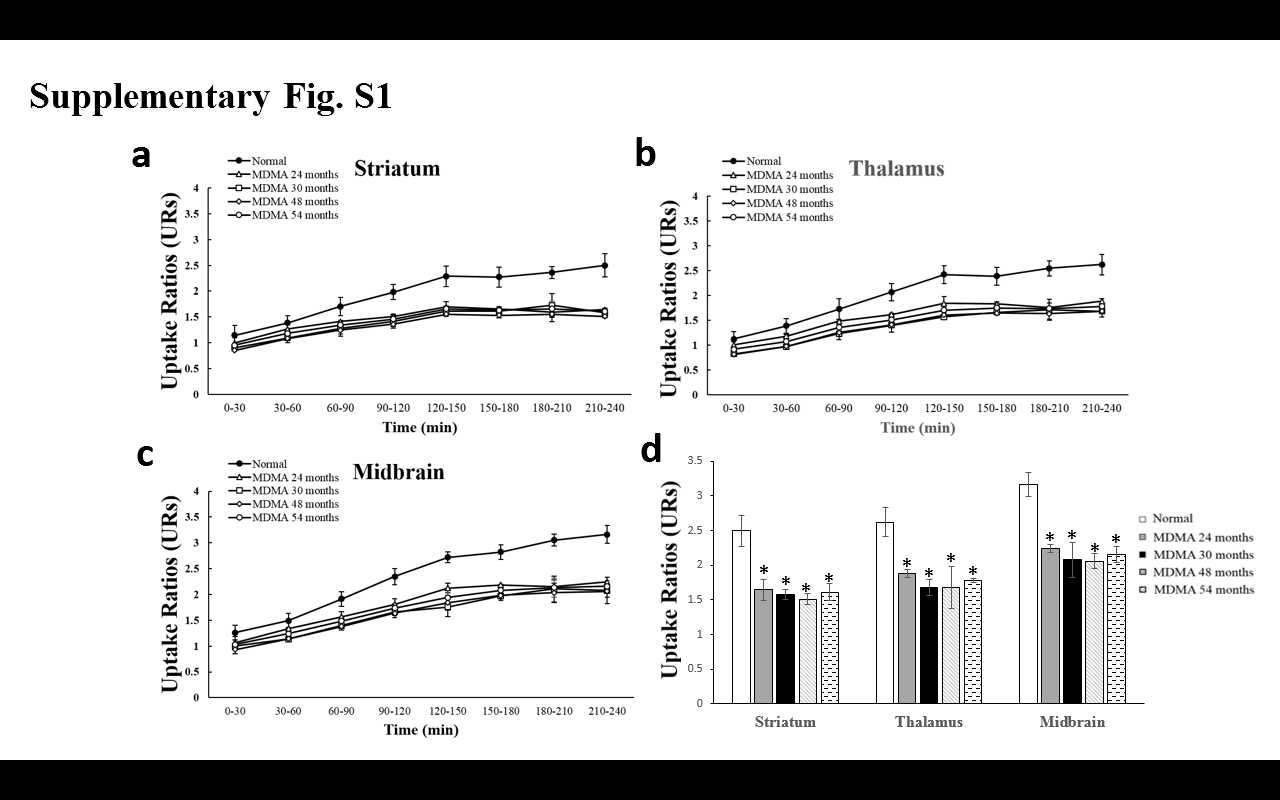


Time-course of mean URs in (a) striatum, (b) thalamus and (c) midbrain at different time points in normal and MDMA groups. (d) URs of [123I]-ADAM SPECT 210-240 min. post-injection in various brain regions of normal and MDMA-treated monkeys at 24, 30, 48 and 54 months. These results are presented as mean ± S.D. *, P<0.05 when compared with normal group.

**Supplementary Figure S2**


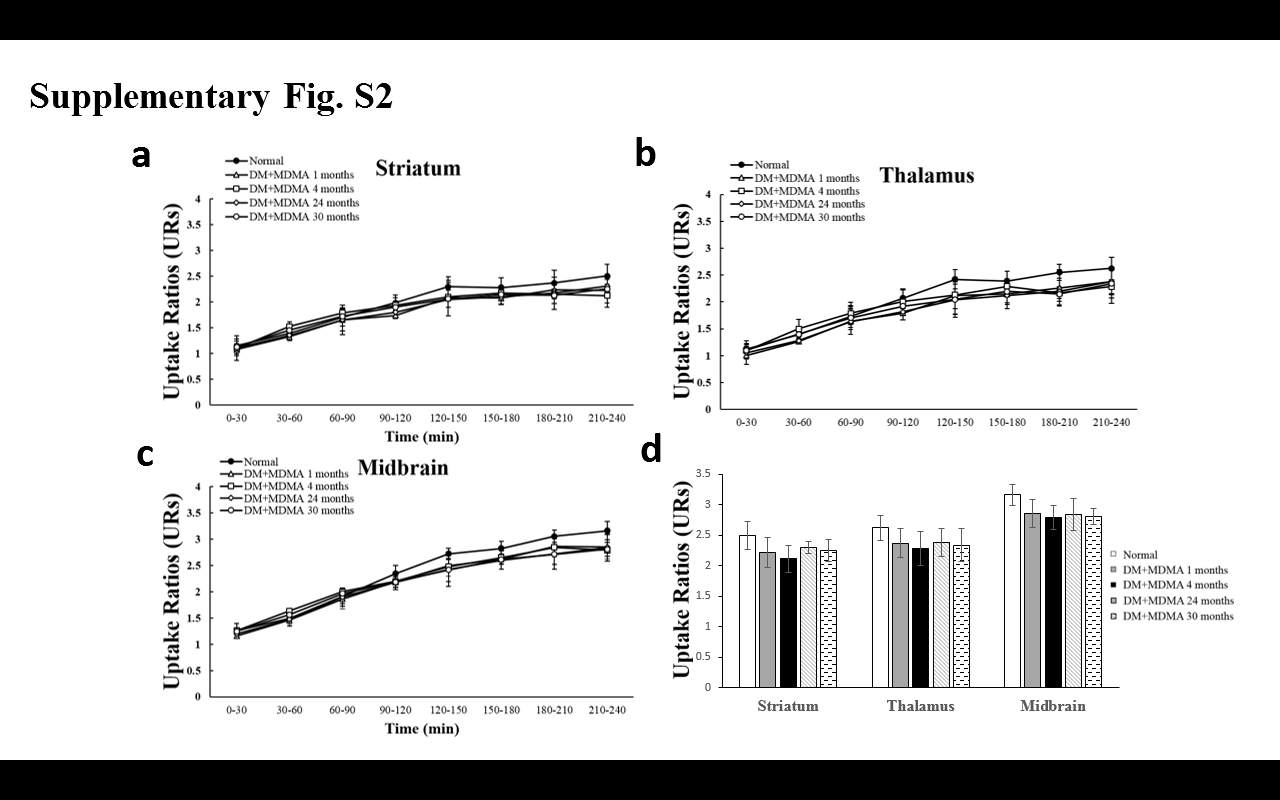


Time-course of mean URs in (a) striatum, (b) thalamus and (c) midbrain at different time points in normal and DM+ MDMA groups. (d) URs of [123I]-ADAM SPECT 210-240 min. post-injection in various brain regions of normal and MDMA/DM-treated monkeys at 1, 4, 24 and 30 months.

**Supplementary Table S1**


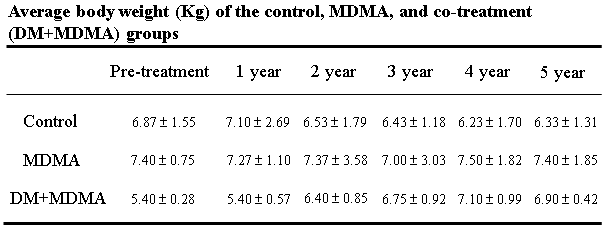


**Supplementary Table S2**


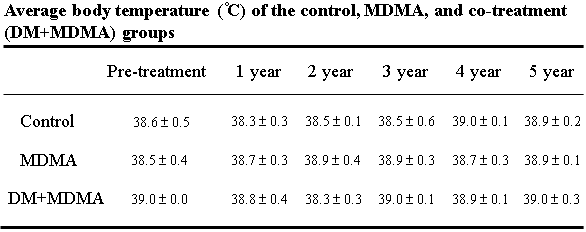

Supplement: Supplementary Dataset 1 [file srep38695-s1.doc]
